# Physical activity and physical activity promotion in Germany – An overview

**DOI:** 10.25646/13557

**Published:** 2025-11-26

**Authors:** Sven Messing, Leonie Birkholz, Julian Resch, Johannes Brandl, Eva Lorenz, Karim Abu-Omar, Wolfgang Geidl, Antonina Tcymbal, Peter Gelius, Klaus Pfeifer

**Affiliations:** 1 University of Limerick, Physical Activity for Health Research Centre, Health Research Institute, Department of Physical Education and Sport Sciences, Limerick, Ireland; 2 Friedrich-Alexander-Universität Erlangen-Nürnberg, Department of Sport Science and Sport, Erlangen, Germany; 3 University of Lausanne, Institute of Sports Science, Lausanne, Switzerland

**Keywords:** Physical activity, Physical activity promotion, Good practice, Effectiveness, Reach, Evaluation, Integrated knowledge translation

## Abstract

**Background:**

Regular physical activity is essential for health, yet a large proportion of the German population remains inactive, with significant health and economic consequences. As physical activity promotion spans multiple settings and political sectors, systematic overviews of available data on behaviour and promotion practices are often lacking.

**Methods:**

This article draws on four policy briefs on physical activity promotion in Germany published by the Federal Ministry of Health (2022 – 2024). Data on physical activity behaviour (secondary analysis) and promotion practices across sectors (mixed methods approach) were analysed. A distinction was made between good practice (projects with proven effectiveness) and routine practice (large-scale programmes).

**Results:**

Between 1993 and 2024, eleven institutions from different political sectors collected data on physical activity behaviour in 23 larger studies. Current data show lower activity levels among older adults, women, socioeconomically disadvantaged groups, and individuals living with a non-communicable disease. In total, 43 good practice projects and 88 routine practice measures were identified. While all good practice projects demonstrated effectiveness, this was true for only 11 % of routine practices. Good practice projects were less likely to reach at least 100,000 people (12 %) compared to routine practice (25 %), and were more often limited to less than five years (33 % vs. 9 %).

**Conclusions:**

Physical activity promotion is an intersectoral challenge requiring stronger structures and shared responsibility. To increase population-level impact, the reach of good practice should be expanded and the effectiveness of routine practice evaluated more systematically. The planned establishment of a National Competence Centre for Physical Activity Promotion offers a key opportunity to advance these goals.

## 1. Introduction

Regular physical activity (PA) is essential for good health. Over the course of their lives, individuals who engage in physical activity can expect a wide range of physical, mental and social health benefits [1–3]. However, it is well established that a significant proportion of the global population does not reach recommended levels of PA. In 2022, 31.3 % of adults worldwide were classified as physically inactive – an increase from 23.4 % in 2000 and 26.4 % in 2010 [4]. Among young people aged 11 to 17, the situation is even more concerning: in 2016, 81.0 % were reported to be insufficiently active [5]. Scientific modelling studies have linked physical inactivity to premature mortality [6] and estimate its global economic burden to range between 47.6 to 67.5 billion USD annually [7, 8]. Notably, physical inactivity is also a social issue. Research consistently shows that individuals with lower income, lower levels of education, a migrant background, or non-communicable diseases are less likely to be physically active compared to other population groups [9–12].

Given these multifaceted challenges, the importance of promoting physical activity has steadily increased in recent decades. At the international level, this is reflected in key policy documents such as the World Health Organization’s (WHO) Global Action Plan on Physical Activity (2018) [13], the Council of the European Union’s Recommendations on Health-Enhancing Physical Activity across Sectors (2013) [14], and ISPAH’s ‘Eight Investments That Work for Physical Activity’, which emphasises the relevance of various settings and sectors for promoting physical activity (e. g. sport, health, transport, school) [15]. In Germany, several initiatives have been adopted. These include the publication of National Recommendations for Physical Activity and Physical Activity Promotion in 2016/2017 [16], as well as the adoption of ‘IN FORM – Germany’s National Initiative to promote Healthy Diets and Physical Activity’ in 2008, which was further developed in 2021 [17, 18].

Since 2022, the Federal Ministry of the Interior and the Federal Ministry of Health have jointly organised two Physical Activity Summits. In addition, the Ministry convened a round table on ‘Physical Activity and Health’, bringing together representatives from various political levels and sectors. In preparation for these initiatives, it became evident that essential information to support the political process – particularly regarding the current state of physical activity promotion in Germany – was either unavailable or had not been systematically compiled. To address this gap, the Federal Ministry of Health commissioned the development of ‘policy briefs on physical activity promotion in Germany’, produced with the involvement of the authors of this article. These policy briefs contain data on prevalence rates as well as an inventory of physical activity promotion practices for four population groups: children and adolescents [19], adults aged 18 to 64 [20], older adults aged 65 and over [21] and adults aged 18 and over a non-communicable disease [22]. Additional research and analysis were conducted for the purpose of this article. All policy briefs follow a consistent structure, enabling comparison and synthesis of findings across population groups.

This article aims to provide a comprehensive overview of physical activity and its promotion in Germany. It addresses four research questions: (1) What is the current state of knowledge regarding physical activity behaviour among the German population? (2) Which scientifically evaluated projects with demonstrated effectiveness (‘good practice’) can be identified through existing databases? (3) What physical activity promotion activities are routinely implemented in Germany (‘routine practice’)? and (4) To what extent do good and routine practices differ in terms of effectiveness, reach and duration? As this article is intended to provide an overview, readers are referred to the policy briefs published by the Federal Ministry of Health [19–22] and other scientific publications [23, 24] for detailed information on methods and results.

**Key messages**

► In Germany, a significant proportion of the population is insufficiently physically active – particularly older adults, women, individuals from socioeconomically disadvantaged backgrounds, and those living with a non-communicable disease.
► Although numerous data sources on physical activity behaviour exist, methodological differences make it challenging to analyse trends over time.
► A total of 43 projects have been identified as good practice with proven effectiveness, but most have limited reach.
► In contrast, 88 routine practice measures are well integrated into existing structures and reach a substantial number of individuals, yet they have rarely been scientifically evaluated.
► To ensure that physical activity promotion is both effective and sustainable, stronger collaboration between research, policy and practice is essential.

## 2. Methods

The methodological approach used to develop the policy briefs was co-produced by researchers from the WHO Collaborating Centre for Physical Activity and Public Health and staff of the Federal Ministry of Health. This collaborative process – referred to in scientific literature as ‘integrated knowledge translation’ – actively involves ‘knowledge users’ in the research process, thereby enhancing the likelihood that research findings will be applied in policy and/or practice [25]. At the outset, no existing monitoring tool met the political need for a comprehensive overview of relevant aspects of physical activity and its promotion in Germany. Consequently, a new tool had to be developed. This instrument, known in the scientific literature as the TARGET:PA tool, is described in detail elsewhere [26]. Political consideration influenced not only the design of the tool but also the timeframe available for data collection and analysis. The tool was first piloted for children and adolescents [23] and then minimally adapted for other population groups. As a result, the methodological approach varies slightly across groups. To ensure consistency in the presentation of findings across all four population groups, additional research and analyses were conducted specifically for this article.

### 2.1 Data on physical activity behaviour in Germany

Datasets on physical activity behaviour were identified through an existing review [11], a study on physical activity and sedentary behaviour among children and adolescents in Germany [27], as well as additional searches in scientific databases and on the websites of relevant organisations. For each dataset, key information was summarised in tabular form, including the responsible institution, age range of respondents, years and frequency of data collection, and sample sizes.

To enable a comparative analysis, variables related to physical activity were extracted from all identified datasets. For adults (aged 18 – 64) and older adults (aged 65+), the analysis focused on the most comparable indicators: the proportion of the population that is not (a) participating in sports or (b) engaging in physical activity of at least moderate intensity.

Current data on physical activity levels among children and adolescents are derived from the German Health Interview and Examination Survey for Children and Adolescents (KiGGS [28]). For the other three population groups, data come from the nationally representative study German Health Update (GEDA) [29]. The reported values reflect compliance with WHO’s 2010 physical activity guidelines [30]. For children and adolescents aged 5 to 17, this corresponds to at least 60 minutes of moderate- to vigorous-intensity physical activity per day. For adults, the recommendation is at least 150 minutes of moderate-intensity physical activity per week, 75 minutes of vigorous-intensity physical activity, or an equivalent combination of both.

### 2.2 Good practice of physical activity promotion in Germany

Good practice refers to approaches that have been shown to produce positive outcomes – i. e. whose effectiveness has been scientifically demonstrated – and can therefore be recommended as exemplary models [31]. Examples of good practice in physical activity promotion in Germany were identified through existing project databases. Relevant databases were selected based on a memorandum from the Federal Centre for Health Education on evidence-based prevention and health promotion [32], a previous study on good practice examples in Germany [33] and additional research. In total, ten databases were identified:

► Network Prevention, Bavarian Centre for Prevention and Health Promotion [34]
► Green List Prevention, Lower Saxony State Prevention Council [35]
► Good Practice Database, Collaborative Network for Equity in Health [36]
► Project database of the German Education Server [37]
► Project database of the State Institute for Health and Work of North Rhine-Westphalia [38]
► Project database ‘Healthy and active ageing’, Federal Institute of Public Health [39]
► Project database ‘IN FORM’ [40]
► Project database of the initiative ‘Impulsgeber Bewegungsförderung’, Federal Institute of Public Health [41]
► SPOFOR Database, Sport Information Portal SURF, Federal Institute for Sport Science (BISp) [42]
► Knowledge for Healthy Settings, GKV Alliance for Health [43]

The databases were analysed for children and adolescents in 2021 and – due to the Federal Ministry of Health’s publication schedule for the individual policy briefs – for the other population groups in 2023 and 2024. Because of differences in timing and thematic focuses, some databases mentioned were analysed only for specific population groups.

In total, 1,061 potentially relevant projects were identified across the databases. An additional 23 projects targeting adults with non-communicable diseases were identified through a stakeholder survey. Of the 1,084 projects reviewed, 574 were excluded due to duplication or lack of relevance (i. e., no focus on promoting physical activity). Among the remaining 510 projects, 43 had documented evidence of effectiveness and were therefore classified as good practice.

For adults with non-communicable diseases, a supplementary survey was conducted in 2024 to identify further projects. Stakeholders were recruited through (a) the interdisciplinary working group on physical activity-related health services research of the German Network Health Service Research, (b) medical and physical activity-related professional associations and self-help groups, and (c) other organisations involved in physical activity promotion in Germany. Seventeen experts participated in the survey, with representation from politics (29.4 %), science (23.5 %), practice (23.5 %) and other sectors (23.5 %). Most respondents were affiliated with the health sector (76.4 %), followed by sport (11.7 %).

### 2.3 Routine practice of physical activity promotion in Germany

Routine practice refers to all activities that are implemented at large scale in Germany to promote physical activity – whether driven by legal regulations, funding mechanisms or the initiative of organisations. This analysis primarily focused on practices at the national level.

To identify routine practice, a range of methods was employed. For children and adolescents, six semi-structured expert interviews were conducted in 2021 as part of the pilot study. Experts represented various sectors and settings, including family and home, early childhood education and care, school, sport, healthcare, transport, and urban planning. For adults, older adults and adults with non-communicable diseases, routine practice was identified through web-based searches conducted in 2023 and 2024. This included reviewing the websites of 64 relevant organisations involved in physical activity promotion in Germany and targeted Google searches. Routine practice was selected based on the following criteria: (a) a clear focus on behaviour- or context-related physical activity promotion, (b) institutional anchoring within an organisation’s structure, and (c) potential for broad population-level reach and public health impact.

To validate and supplement the findings, two online surveys were conducted among stakeholders in physical activity promotion (one for adults and older adults, and another for adults with non-communicable diseases). For the first survey, participants were recruited from a list of relevant organisations from various sectors and settings (e.g. organisations implementing routine practice). A total of 45 individuals responded, with representation from practice (22.2 %), science (19.4 %), politics (16.7 %), and other sectors (41.7 %). Most respondents were affiliated with the health sector (48.9 %), followed by sport (13.3 %) and nursing homes (8.9 %). Seventeen individuals participated in the second survey for adults with non-communicable diseases (see section 2.2), i.e. a total of 62 experts responded to the two surveys. Both surveys contained a tabular overview of routine practice for the respective population group as well as questions (a) to identify further measures and (b) to validate the information presented in the tables.

### 2.4 Effectiveness, reach and duration of physical activity promotion practices

To compare good and routine practices in physical activity promotion, suitable criteria were selected based on the REAIM framework [44] and a study on quality criteria for interventions promoting physical activity [45]. Through the co-production process, three criteria were identified as particularly relevant:

► Effectiveness: Evidence of effects on physical activity behaviour and/or related indicators
► Reach: Total number of individuals reached
► Duration: Length of time the project or measure has been implemented

For projects classified as good practice, information on effectiveness, reach and duration was extracted from project databases, reports, scientific publications, and websites. For routine practice, relevant data were gathered through online searches, including project websites and targeted Google searches. The following categories were used for the analysis:

► Effectiveness: yes/no
► Reach: Less than 10,000 people/10,000 to less than 100,000 people/100,000 to less than 1,000,000 people/1,000,000 people or more/Data not available
► Duration: Less than 5 years/5 to less than 10 years/10 to less than 20 years/20 years or more/Data not available

## 3. Results

### 3.1 Data on physical activity behaviour in Germany

A total of 23 large-scale studies that collect data on physical activity behaviour in Germany – most of which are population-representative – were identified. These studies were conducted by eleven different institutions between 1993 and 2024, in some cases involving multiple survey waves. The studies cover various age groups and indicators. While some collect data on physical activity across different domains, others focus specifically on sports and leisure activities or active mobility. Sample sizes vary considerably across studies, ranging from 850 to 421,000 respondents per survey (Table 1).

**Figure 1:**
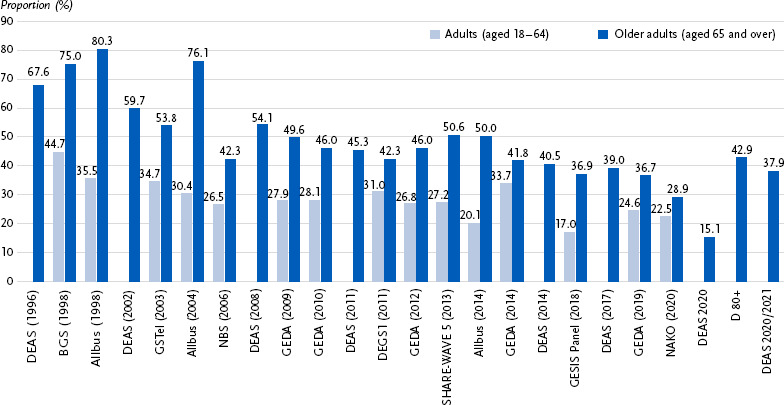
Percentage of individuals who do not engage in any sporting activity – results from 24 surveys on physical activity behaviour Explanation of study abbreviations: see Table 1

**Figure 2:**
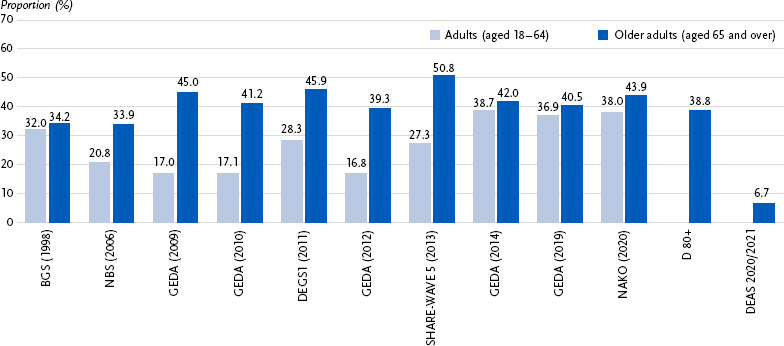
Percentage of individuals who never or almost never engage in physical activity of at least moderate intensity– results from 12 surveys on physical activity behaviour Explanation of study abbreviations: see Table 1

Due to the varying indicators and survey methodologies of the identified studies, comparative analyses are only possible to a limited extent. However, two commonly used indicators are included in several studies: (a) participation in sport and (b) total physical activity. Figure 1 presents data from 24 surveys, showing that the proportion of adults who do not participate in any sporting activities ranges from 17.0 % to 44.7 %, while for older adults, the range is broader – from 15.1 % to 80.3 %. Figure 2 displays results from 12 surveys, comparing the percentage of individuals who never or almost never engage in physical activity of at least moderate intensity. Among adults, this proportion ranges from 16.8 % to 38.7 %, and among older adults, from 6.7 % to 50.8 %.

**Figure 3:**
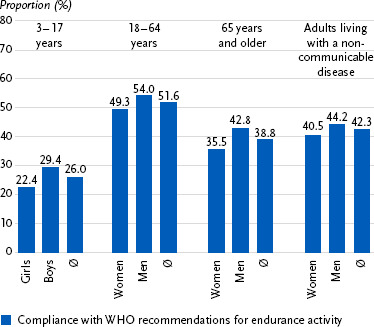
Percentage of individuals meeting WHO’s Physical Activity Guidelines by gender and population group – based on KiGGS (n = 6,532 girls, n = 6,449 boys) and GEDA (n = 11,959 women, n = 10,687 men) studies. Sources: KiGGS Wave 2, GEDA 2019/2020-EHIS

Figure 3 illustrates the proportion of individuals meeting WHO’s Physical Activity Guidelines across four population groups: children and adolescents, adults, older adults, adults living with a non-communicable disease. This indicator is particularly important as it predicts the risk of non-communicable diseases and enables international comparisons. The figure highlights clear gender and age differences: across all age groups, boys/men are more physically active than girls/women, and older adults (65+) are less active than adults aged 18 to 64. Moreover, studies confirm the influence of social determinants on physical activity behaviour in Germany. Lower income, lower educational attainment, and a migrant background are associated with reduced participation in sports and lower levels of at least moderate-intensity physical activity. Girls appear to be more affected by social disadvantage than boys [11, 28].

### 3.2 Good practice in promoting physical activity in Germany

The 43 good practice projects are distributed as follows across population groups, settings and sectors:

► Children and adolescents (n = 22): Good practice was identified almost exclusively in the settings early childhood education and care (n = 9) and school (n = 12). Another project aimed at promoting active transport to kindergartens and schools was assigned to the transport sector (n = 1).
► Adults (n = 6): Good practice was identified for the workplace (n = 4) and community (n = 2) settings. These projects are aimed at potentially disadvantaged population groups such as the long-term unemployed (n = 2), women in difficult life situations (n = 2) and people with disabilities (n = 1), as well as occupational groups with high physical demands (nursing staff; n = 1).
► Older adults (n = 7): Good practice was identified for retirement and nursing homes (n = 3) and community (n = 4). One of the projects in retirement homes is explicitly aimed at nursing staff, thus also addressing adults in the workplace. The community projects are mainly aimed at specific population groups such as people at high risk of dementia (n = 1), patients at risk of falling (n = 1) and older people with a migrant background (n = 1).
► Adults living with a non-communicable disease (n = 8): All good practice projects for this population group were assigned to the health sector (n = 8). Some of these projects are aimed at people with specific diseases such as cancer (n = 2), multiple sclerosis (n = 1), dementia (n = 1) and obesity (n = 1). Two other projects target patients in inpatient rehabilitation (n = 1) or following inpatient rehabilitation (n = 1). One of the projects is designed as an extra-occupational measure to ensure employability and thus overlaps with the workplace (n = 1).

### 3.3 Routine practice of physical activity promotion in Germany

A total of 88 routine practices were identified, specified by 104 specific examples of measures implemented by stakeholders promoting physical activity in Germany. The findings can be summarised as follows:

► Children and adolescents (n = 27): Routine practice was identified in family and home (n = 5), early childhood education and care (n = 6), school (n = 7), sport (n = 3), health (n = 1) and transport (n = 5). Examples include parent-child gymnastics, physical education classes, partnerships between kindergartens or schools and sports clubs, exercise referrals during preventive medical check-ups, and traffic-calming measures near schools.
► Adults (n = 35): Routine practice was found in sport (n = 9), workplace (n = 7), community (n = 7), transport (n = 6), health (n = 2), mass media campaigns (n = 1) and other areas (n = 3). Examples include quality seals for health promotion in sports clubs, workplace physical activity initiatives as part of occupational health management, free sports equipment rental in municipalities, bicycle-sharing systems and prevention courses offered by health insurers.
► Older adults (n = 16): Routine practice was identified in retirement and nursing homes (n = 4), sport (n = 3), community (n = 3), home environment (n = 2), transport (n = 1) and other areas (n = 3). Examples include fall prevention programmes, health-oriented sports courses, multi-generational parks/playgrounds, activating home visits and cycling safety training.
► Adults with non-communicable diseases (n = 10): Routine practice was concentrated in health (n = 8) and sport (n = 2). Measures include primary care interventions (e.g. disease management programmes), medical rehabilitation (e.g. sports and exercise therapy), and online databases listing relevant physical activity and sports programmes.

### 3.4 Effectiveness, reach and duration of physical activity promotion practices

Good practice (n = 43) and routine practice (n = 88) differ substantially in terms of effectiveness, reach and duration. However, these comparisons are limited by gaps in data availability. Information on effectiveness was available for 53 of the 131 projects and measures (43 good practice/10 routine practice), reach is known for 54 (31/23) and duration for 106 (42/64).

Effectiveness was demonstrated for all 43 good practice projects (100 %), as this was the inclusion criterion. However, the type of evidence varied in methodology (qualitative vs. quantitative) and outcomes (e.g. increased physical activity, improved motor performance, enhanced knowledge). For routine practice, effectiveness was documented for only 10 of 88 measures (11 %), with no corresponding data for the remaining 78 measures (89 %).

**Figure 4:**
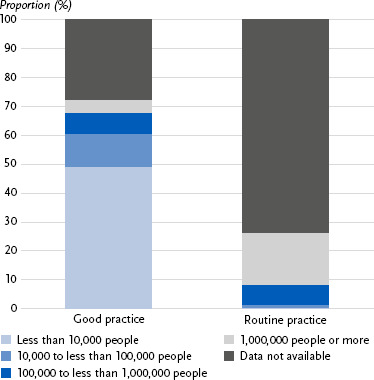
Reach (number of individuals reached) for good and routine practices in physical activity promotion

Reach also varies considerably (Figure 4). Among good practice projects, 60 % reached less than 100,000 people, while 12 % reached more than 100,000. For routine practice, only 1 % reached less than 100,000 people, whereas 25 % reached more than 100,000. Data on reach were unavailable for approximately three quarters of routine practice measures (74 %) and about a quarter of good practice projects (28 %).

**Figure 5:**
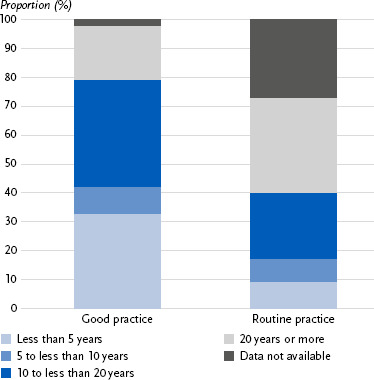
Duration of good and routine practices in physical activity promotion

Differences were also observed in duration (Figure 5). While 33 % of good practice projects lasted less than five years, this was the case for only 9 % of routine practice measures. Missing data was more common for routine practice (27 %) than for good practice (2 %).

## 4. Discussion

This article provides an overview of key data on physical activity and physical activity promotion in Germany. In total, 23 studies on physical activity behaviour, 43 good practice projects, and 88 routine practice measures were analysed.

### 4.1 Data on physical activity behaviour in Germany

Current data indicate that a substantial proportion of the population in Germany is physically inactive. Moreover, the data reveal significant differences in terms of age (older age groups are less active), gender (in most age groups, women are less active than men), socioeconomic status (lower income and lower education correlate with lower levels of physical activity) and health status (individuals with non-communicable diseases are less active).

Determining whether physical activity levels in Germany have changed over recent decades remains challenging. There are indications that at least the proportion of individuals not participating in sports has declined, but overall trends are uncertain. This is surprising given the large number of high-quality surveys covering different age groups. However, several methodological challenges explain this uncertainty.

First, measuring physical activity at the population level is inherently complex. The limitations of self-report questionnaires are well documented [46]. Respondents often fail to recall recent activity accurately – particularly its intensity – or misjudge it. Social desirability bias may lead to over-reporting of activity, and questions about physical activity are often cognitively demanding and difficult to understand [47]. These issues have been exacerbated by the paradigm shift in the mid-1990s from focusing solely on sport to capturing health-enhancing physical activity across multiple domains (e.g. work, transport).

Second, the proportion of individuals meeting WHO’s Physical Activity Guidelines is a key indicator in health monitoring to ensure international comparability [48]. However, linking measurement to these recommendations has unintended consequences: even minor adjustments to the guidelines necessitate changes to measurement instruments, reducing comparability over time.

Third, competing measurement methods add complexity. Accelerometry – using motion sensors – has long been routine in smaller, often clinical studies, offering high quality, objective data without the biases of memory, social desirability and understanding. Despite numerous pilot studies, accelerometry has not been widely adopted in large-scale population surveys due to logistical challenges, technical limitations (e.g. capturing cycling) and higher costs [49]. Some of these issues may be mitigated by leveraging smart-phone-based accelerometers [50].

### 4.2 Physical activity promotion practices in Germany

The analysis reveals substantial differences between good and routine practices in terms of effectiveness, reach and duration. While good practice projects demonstrate effectiveness by definition, they often have limited reach. Conversely, routine practice typically reaches a much larger number of individuals and is implemented over longer periods of time; however, effectiveness is rarely evaluated.

Notably, more than half of the identified good practice projects target children and adolescents. This is encouraging, as childhood and adolescence are widely regarded as a critical period for promoting physical activity, with early habits influencing lifelong behaviour [51–53]. This focus may also reflect the relative ease of conducting intervention studies with control groups in structured settings such as early childhood education and school – as well as workplaces, nursing homes, and healthcare facilities, where many other good practice projects are situated. However, some researchers criticize this type of research – often based on randomised controlled trials -for favouring ‘micro-interventions’ and diverting attention from the need for structural and institutional change [54, 55]. Nevertheless, population-level impact appears achievable if effective projects are successfully scaled and structurally embedded. Germany offers positive examples of successful scaling, particularly in the education sector: The ‘Klasse 2000’ programme, which includes a physical activity component, has reached 2.3 million children since 1991; in the 2023/2024 school year, 15.3 % of all school classes (n = 22,373) participated [56]. Similarly, the ‘fit4future’ initiative has reached approximately 1.8 million children and adolescents in daycare centres and schools since 2016 [57]. However, not all good practice projects can be scaled up in this way, underscoring the importance of understanding contextual factors and success criteria for scaling up [58, 59].

Successfully scaled projects combine the strengths of good practice (proven effectiveness) and routine practice (broad reach and structural anchoring). Examples include the ‘Lübeck World of Movement Model’, designed for older adults requiring care and now implemented in over 100 facilities nationwide [60, 61], and the ‘AlltagsTrainingProgramm’ (Everyday Training Programme), which promotes physical activity in everyday life through prevention courses and as a permanent offer [62, 63]. Both originated as scientifically evaluated pilot programmes and are now embedded within the Federal Institute for Public Health’s ‘ Healthy & Active Ageing’ initiative. However, such cases remain exceptions: most routine practice measures lack evaluation, leaving their effectiveness unknown. This raises the question of whether systematic evaluation of routine practice should be prioritised to inform evidence-based improvements. Given the extensive reach of existing structures – such as over 28 million sport club memberships [64], more than eleven million school-aged children and adolescents [65] and an average of nine to ten physician visits per person annually [66] – leveraging these systems offers significant potential. Even modest annual increases in the reach or effectiveness of routine practice could yield greater population-level impact than continuously developing new pilot projects. In this context, greater emphasis on structural and policy-related interventions is warranted, supported by co-production processes that foster collaboration between policymakers, practitioners and researchers [25, 67]. Recent national research increasingly explores the suitability of such approaches for sustainable development and implementation of physical activity promotion measures across divers settings and sectors, including community, workplace, education and healthcare [58, 68,–70].

Internationally, these issues are framed under concepts such as ‘evidence-to-practice’ (good practice) and ‘practice-to-evidence’ (routine practice), among others [59]. In a special issue of ‘The Lancet’, Rodrigo Reis and colleagues emphasised the importance of both perspectives, while cautioning that pilot projects alone are insufficient. They advocate for research on scaling up and the implementation of effective policy and practical measures to promote physical activity [59]. The authors further argue that such a shift in the research agenda requires support from funding bodies, recommending flexible funding mechanisms that enable the generation of practice-based evidence – for example, through evaluation of successfully scaled projects [59].

### 4.3 Limitations

Several limitations of this study should be noted. First, data for the different policy briefs were collected at different times between 2021 to 2024. Although a uniform structure was applied, methodological detail varied. For example, to identify routine practice for children and adolescents, expert interviews were conducted first and then validated through online research. For the other three population groups, these steps were reversed, and interviews were replaced by an online survey. While this methodological refinement following the pilot study improved the scope and quality of results, it complicates direct comparisons across population groups. Further limitations arise from gaps in data availability, which future surveys may address. These include missing data on physical activity behaviour (e.g. for children under three years of age or individuals with rare or multiple diseases) and on physical activity promotion practices (e.g. effectiveness, reach and duration). Additionally, the policy briefs focused primarily on the national level; consequently, routine practice at regional and local levels were only selectively considered, despite their relevance for physical activity promotion [71]. Finally, the research strategy may have resulted in some good practice projects being overlooked, as identification relied on selected databases. Future work could complement this study with a systematic review of the scientific literature to capture additional projects.

### 4.4 Conclusions

The findings underscore the intersectoral nature of physical activity promotion: data on physical activity behaviour is currently collected by eleven institutions across diverse political sectors. Real-life approaches are central to physical activity promotion, spanning settings from early childhood education and schools to retirement and nursing homes. Strengthening cross-sector dialogue is therefore essential. The Federal Ministry of Health initiated such a dialogue in 2022 through the ‘Round Table on Physical Activity and Health’, aiming to leverage the health benefits of physical activity across the population [72]. The consensus paper published in early 2024 provides a crucial foundation for the political process, outlining concrete measures to advance physical activity promotion in Germany [72].

One key measure is the update and further development of the National Recommendations for Physical Activity and Physical Activity Promotion. The current recommendations, published in 2016 [16], have had broad political and social impact, triggering numerous national activities to promote physical activity. The update is already being implemented: since early 2025, the Federal Ministry of Health funds a corresponding project, which includes a stakeholder participation process [21]. In parallel, the Federal Ministry of Education and Research funds the IMPAQT project, focusing on policy measures to promote physical activity and health equity [73].

Additionally, the Federal Ministry of Health reaffirmed its commitment at the round table to establish a National Competence Centre for Physical Activity Promotion [72]. This initiative builds on a 2021 Federal Cabinet decision to create a central structure for physical activity promotion [18], which has yet to be implemented. As an intermediary organisation, such a centre could facilitate knowledge transfer from research to practice – for example by initiating, moderating and evaluating real-life co-production processes at various political levels [74, 75]. Coordinating activities across political sectors could be a key responsibility, alongside fostering strategic collaboration between science, policy and practice on issues such as scaling up good practice and evaluating routine practice.

## Figures and Tables

**Table 1:** Datasets on physical activity behaviour in Germany

Institution	Dataset	Age range	Survey	Sample size (*rounded*)
Federal Ministry of Transport	Mobility in Germany (MiD)	From 0 years	4 surveys 2002 – 2023	60,000 – 421,000
German Youth Institute	Growing up in Germany (AID:A)	Varies, generally from 0 to 32 – 37 years of age	6 surveys 2009 – 2023	1,500 – 22,000
	AID:A Migration	0 – 32 years	2019	1,900
	AID:A NRW+	0 – 32 years	2019	2,400
	AID:A Corona	1 – 33 years	2020	1,000
	Media, culture and sport among young people (MediKuS)	9 – 24 years	2011 – 2012	4,900
German Centre of Gerontology	German Ageing Survey (DEAS)	40 years and older	9 surveys 1996 – 2023	4,800 – 10,300
	Old age in Germany (D80+)	From 80 years	2020/2021	10,600
GfK Society for Consumer Research	Non-mover study (NBS)	From 10 years	2006	10,600
Karlsruhe Institute of Technology	Motorik-Module (MoMo) of the KiGGS study	4 – 17 years	4 cross-sectional surveys 2003 – 2022	4,200 – 5,100
		4 – 17 years (at the start of the study)	1 longitudinal survey 2003 – 2022	850 – 4,500
Leibniz Institute for Educational Trajectories	National Education Panel Study (NEPS)	From 0 years	Since 2008	70,000
GESIS – Leibniz Institute for the Social Sciences	Allbus	From 18 years of age	3 surveys 1998 – 2014	2,900 – 3,500
	GESIS Panel 2018	Aged 18 years and over	2013 – 2018	7,600
SHARE^1^ BERLIN Institute (SBI)	Survey of Health, Ageing and Retirement in Europe (SHARE-WAVE 1)	From 50 years	9 surveys 2004 – 2022	5,800 in SHARE-WAVE 5*
German National Cohort (NAKO)	German National Cohort	From 20 years	Since 2014	205,000
Robert Koch Institute	German National Health Interview and Examination Survey 1998 (GNHIES98)	18 – 79 years	1997 – 1999	7,100
	German Health Interview and Examination Survey for Adults (DEGS)	18 – 79 years	2008 – 2011	8,000
	German Health Update (GEDA)	From 15/18 years	9 surveys 2009 – 2024	5,000 – 33,100
	GEDA Fokus	From 18 years	2021 – 2022	6,000
	Study on Health of Older People in Germany (Gesundheit 65+)	From 65 years	2021 – 2023	3,700
	Telephone Health Survey (GSTel03)	From 18 years	2003	8,300
	German Health Interview and Examination Survey for Children and Adolescents (KiGGS)	0 – 17/18 years	3 cross-sectional surveys 2003 – 2017	12,400 – 17,600
		0 – 17 years (at the start of the study)	1 longitudinal survey 2003 – 2017	10,900 – 17,600
World Health Organization (WHO)	Health Behaviour in School-Aged Children (HBSC)	11 – 15 years	8 surveys 1993 – 2022	4,300 – 6,500*

*Data not available for all surveys

^1^Survey of Health, Ageing and Retirement in Europe

## References

[1] Joisten C Pfeifer K Vogt L. Wirkungen auf physische Gesundheit und Wohlbefinden. In: Thiel A Tittlbach S Sudeck G Wagner P Woll A, editors. Handbuch Bewegungsbezogene Gesundheitsförderung. Schorndorf: Hofmann-Verlag GmbH & Co. KG; 2023. p. 156–73.

[2] Pahmeier I Kleinert J Sudeck G Wunsch K. Wirkungen auf psychische Gesundheit und Wohlbefinden. In: Thiel A Tittlbach S Sudeck G Wagner P Woll A, editors. Handbuch Bewegungsbezogene Gesundheitsförderung. Schorndorf: Hofmann-Verlag GmbH & Co. KG; 2023. p. 174–92.

[3] Schmid J Sudeck G. Wirkungen auf soziales Wohlbefinden. In: Thiel A Tittlbach S Sudeck G Wagner P Woll A, editors. Handbuch Bewegungsbezogene Gesundheitsförderung. Schorndorf: Hofmann-Verlag GmbH & Co. KG; 2023. p. 193–202.

[4] Strain T Flaxman S Guthold R Semenova E Cowan M Riley LM. National, regional, and global trends in insufficient physical activity among adults from 2000 to 2022: a pooled analysis of 507 population-based surveys with 5.7 million participants. Lancet Glob Health. 2024;12(8):e1232-e1243. doi:10.1016/S2214-109X(24)00150-5. 38942042 10.1016/S2214-109X(24)00150-5 PMC11254784

[5] Guthold R Stevens GA Riley LM Bull FC. Global trends in insufficient physical activity among adolescents: a pooled analysis of 298 population-based surveys with 1.6 million participants. Lancet Child Adolesc Health. 2020;4(1):23–35. doi:10.1016/S2352-4642(19)30323-2. 31761562 10.1016/S2352-4642(19)30323-2 PMC6919336

[6] Lee I-M Shiroma EJ Lobelo F Puska P Blair SN Katzmarzyk PT. Effect of physical inactivity on major non-communicable diseases worldwide: an analysis of burden of disease and life expectancy. Lancet. 2012;380(9838):219–29. doi:10.1016/s0140-6736(12)61031-9. 22818936 10.1016/S0140-6736(12)61031-9 PMC3645500

[7] Ding D Lawson KD Kolbe-Alexander TL Finkelstein EA Katzmarzyk PT van Mechelen W. The economic burden of physical inactivity: a global analysis of major non-communicable diseases. Lancet. 2016;388(10051):1311–24. doi:10.1016/S0140-6736(16)30383-X. 27475266 10.1016/S0140-6736(16)30383-X

[8] Santos AC Willumsen J Meheus F Ilbawi A Bull FC. The cost of inaction on physical inactivity to public health-care systems: a population-attributable fraction analysis. Lancet Glob Health. 2023;11(1): e32-e39. doi:10.1016/S2214-109X(22)00464-8. 36480931 10.1016/S2214-109X(22)00464-8 PMC9748301

[9] Caperchione CM Kolt GS Mummery WK. Physical activity in culturally and linguistically diverse migrant groups to Western society: a review of barriers, enablers and experiences. Sports Med. 2009;39(3): 167–77. doi:10.2165/00007256-200939030-00001. 19290674 10.2165/00007256-200939030-00001

[10] Morseth B Jacobsen BK Emaus N Wilsgaard T Jørgensen L. Secular trends and correlates of physical activity: The Tromsø Study 1979-2008. BMC Public Health. 2016;16(1):1215. doi:10.1186/s12889-016-3886-z. 27912742 10.1186/s12889-016-3886-z PMC5135806

[11] Abu-Omar K Messing S Sarshar M Gelius P Ferschl S Finger J. Sociodemographic correlates of physical activity and sport among adults in Germany: 1997–2018. Ger J Exerc Sport Res. 2021;51(2): 170–82. doi:10.1007/s12662-021-00714-w.

[12] Sudeck G Geidl W Abu-Omar K Finger JD Krauß I Pfeifer K. Do adults with non-communicable diseases meet the German physical activity recommendations? Ger J Exerc Sport Res. 2021;51(2): 183–93. doi:10.1007/s12662-021-00711-z.

[13] World Health Organization (WHO). Global action plan on physical activity 2018-2030: more active people for a healthier world. Geneva, Switzerland: WHO; 2018.

[14] Council of the European Union. Council Recommendation of 26 November 2013 on promoting health-enhancing physical activity across sectors; 2013.

[15] Milton K Cavill N Chalkley A Foster C Gomersall S Hagstromer M. Eight Investments That Work for Physical Activity. J Phys Act Health. 2021;18(6):625–30. doi:10.1123/jpah.2021-0112. 33984836 10.1123/jpah.2021-0112

[16] Rütten A Pfeifer K. Nationale Empfehlungen für Bewegung und Bewegungsförderung. Erlangen: FAU University Press. 2016. 10.1055/s-0042-123346 28399579

[17] Bundesministerium für Ernährung und Landwirtschaft. IN FORM. Deutschlands Initiative für gesunde Ernährung und mehr Bewegung: Nationaler Aktionsplan zur Prävention von Fehlernährung, Bewegungsmangel, Übergewicht und damit zusammenhängenden Krankheiten. 2008 [cited 26.09.2025]. Available from: https://www.bundesgesundheitsministerium.de/fileadmin/Dateien/5_Publikationen/Praevention/Broschueren/IN_FORM_Nationaler_Aktionsplan_zur_Praevention_von_Fehlernaehrung__Bewegungsmangel__UEbergewicht_und_damit_zusammenhaengenden_Krankheiten.pdf.

[18] Bundesministerium für Gesundheit, Bundesministerium für Ernährung und Landwirtschaft. Aktionsplan „Weiterentwicklung IN FORM – Schwerpunkte des Nationalen Aktionsplans zur Prävention von Fehlernährung, Bewegungsmangel, Übergewicht und damit zusammenhängenden Krankheiten ab 2021“. 2021 [cited 26.09.2025]. Available from: https://www.in-form.de/fileadmin/SITE_MASTER/content/Downloads_IN_FORM/weiterentwicklung-aktionsplan-in-form-ab-2021.pdf.

[19] Bundesministerium für Gesundheit. Bestandsaufnahme zur Bewegungsförderung bei Kindern und Jugendlichen in Deutschland (Kurzversion und Langversion). Abu-Omar K Beck F Geidl W Gelius P Grüne E Marzi I. 2022 [cited 26.09.2025]. Available from: https://www.bundesgesundheitsministerium.de/service/publikationen/details/bestandsaufnahme-zur-bewegungsfoerderung-bei-kindern-und-jugendlichen-in-deutschland-langversion.html.

[20] Bundesministerium für Gesundheit. Bestandsaufnahme der Bewegungsförderung bei Erwachsenen (18-64 Jahre) in Deutschland (Kurzversion und Langversion). Abu-Omar K Birkholz L Brandl J Geidl W Gelius P Messing S. 2024 [updated 2024; cited 26.09.2025]. Available from: https://www.bundesgesundheitsministerium.de/service/publikationen/details/bestandsaufnahme-der-bewegungsfoerderung-bei-erwachsenen-18-64-jahre-in-deutschland.html.

[21] Bundesministerium für Gesundheit. Bestandsaufnahme der Bewegungsförderung bei älteren Erwachsenen (ab 65 Jahren) in Deutschland (Kurzversion und Langversion). Abu-Omar K Birkholz L Brandl J Geidl W Gelius P Messing S. 2024 [cited 26.09.2025]. Available from: https://www.bundesgesundheitsministerium.de/service/publikationen/details/bestandsaufnahme-der-bewegungsfoerderung-bei-aelteren-erwachsenen-ab-65-jahren-in-deutschland.html.

[22] Bundesministerium für Gesundheit. Bestandsaufnahme der Bewegungsförderung bei Erwachsenen mit nichtübertragbaren Erkrankungen in Deutschland (Kurzversion und Langversion). Abu-Omar K Birkholz L Brandl J Geidl W Gelius P Messing S. 2024 [updated 2024; cited 26.09.2025]. Available from: https://www.bundesgesundheitsministerium.de/service/publikationen/details/bestandsaufnahme-der-bewegungsfoerderung-bei-erwachsenen-mit-nicht-uebertragbaren-erkrankungen-in-deutschland.html.

[23] Messing S Gelius P Abu-Omar K Marzi I Beck F Geidl W. Developing a policy brief on physical activity promotion for children and adolescents. Front Public Health. 2023;111215746. doi:10.3389/fpubh.2023.1215746. 10.3389/fpubh.2023.1215746 PMC10571038 37841728

[24] Gelius P Messing S Abu-Omar K Marzi I Beck F Geidl W. Collaborative Development of an Instrument to Monitor Physical Activity Promotion Based on Policy-Makers’ Needs – the TARGET:PA Tool. Int J Health Policy Manag. 2025;14:8720. doi: 10.34172/ijhpm.8720. 10.34172/ijhpm.8720 PMC12595577 41966192

[25] Smith B Williams O Bone L the Moving Social Work Co-production Collective. A resource to guide co-producing research in the sport, exercise, and health sciences. Qual Res Sport Exerc Health. 2023;15(2):159–87. doi:10.1080/2159676X.2022.2052946.

[26] Gelius P Messing S Abu-Omar K Marzi I Beck F Geidl W. Collaborative Development of an Instrument to Monitor Physical Activity Promotion Based on Policy-Makers’ Needs – the TARGET:PA Tool. Int J Health Policy Manag. 2025;14:8720. doi: 10.34172/ijhpm.8720. 10.34172/ijhpm.8720 PMC12595577 41966192

[27] Demetriou Y Beck F Sturm D Abu-Omar K Forberger S Hebestreit A. Germany’s 2022 Report Card on Physical Activity for Children and Adolescents. Ger J Exerc Sport Res. 2024;54(2):260–75. doi:10.1007/s12662-024-00946-6.

[28] Finger JD Varnaccia G Borrmann A Lange C Mensink GBM. Physical activity among children and adolescents in Germany. Results of the cross-sectional KiGGS Wave 2 study and trends. J Health Monit. 2018;3(1):23–30. doi: 10.17886/RKI-GBE-2018-023.2. 10.17886/RKI-GBE-2018-023.2 PMC8848914 35586180

[29] Manz K Domanska OM Kuhnert R Krug S. Wie viel sitzen Erwachsene? Ergebnisse der Studie Gesundheit in Deutschland aktuell (GEDA 2019/2020-EHIS). 2022. doi:10.25646/10294.

[30] World Health Organization (WHO). Global recommendations on physical activity for health. Geneva, Switzerland: WHO; 2010. p. 58. 26180873

[31] CHRODIS. Joint Action on Chronic Diseases & Promoting Healthy Ageing across the Life Cycle – Work Package 5: Task 3.: Good Practices in Health Promotion & Primary Prevention of Chronic Diseases. Summary Report. 2015.

[32] de Bock F Dietrich M Rehfuess E. Evidenzbasierte Prävention und Gesundheitsförderung. Memorandum der Bundeszentrale für gesundheitliche Aufklärung (BZgA). Köln: Bundeszentrale für gesundheitliche Aufklärung; 2020.

[33] Henn A Karger C Wöhlken K Meier D Ungerer-Röhrich U Graf C. Identifikation von Beispielen guter Praxis der Bewegungsförderung – Methoden, Fallstricke und ausgewählte Ergebnisse. Gesundheitswesen. 2017;79(S 01):S66-S72. ger. doi:10.1055/s-0042-123697. 28399589 10.1055/s-0042-123697

[34] Bayerisches Zentrum für Prävention und Gesundheitsförderung im Landesamt für Gesundheit und Lebensmittelsicherheit. Netzwerk Prävention [cited 22.04.2025]. Available from: https://www.zpg-bayern.de/netzwerk-praevention.html.

[35] Landespräventionsrat Niedersachsen. Grüne Liste Prävention [cited 22.04.2025]. Available from: https://www.gruene-liste-praevention.de/nano.cms/datenbank/alle.

[36] Gesundheit Berlin-Brandenburg e.V. Praxisdatenbank Gesundheitliche Chancengleichheit [cited 22.04.2025]. Available from: https://www.gesundheitliche-chancengleichheit.de/praxisdatenbank/.

[37] DIPF Leibniz-Institut für Bildungsforschung und Bildungsinformation. Deutscher Bildunsserver: Projekte/ Initiativen zur Bewegungsförderung [cited 22.04.2025]. Available from: https://www.bildungsserver.de/elementarbildung/projekte-initiativen-3673-de.html.

[38] Landeszentrum Gesundheit Nordrhein-Westfalen. Projektdatenbank. 2021.

[39] Bundesinstitut für Öffentliche Gesundheit. Gesund & Aktiv Älter Werden: Projektdatenbank [cited 22.04.2025]. Available from: https://www.gesund-aktiv-aelter-werden.de/fachinformationen/projektdatenbank/recherche-von-angeboten-in-der-projektdatenbank/.

[40] Bundesanstalt für Landwirtschaft und Ernährung. IN FORM: Projektdatenbank [cited 22.04.2025]. Available from: https://www.in-form.de/projekte/projektdatenbank.

[41] Bundesinstitut für Öffentliche Gesundheit. Impulsgeber Bewegungsförderung: Projektsammlung [cited 22.04.2025]. Available from: https://www.gesund-aktiv-aelter-werden.de/impulsgeber-bewegungsfoerderung/projektsammlung/.

[42] Bundesinstitut für Sportwissenschaft. BISp-Datenbanken: Projekt (SPOFOR) [cited 22.04.2025]. Available from: https://www.bisp-surf.de/Search/Results?hiddenFilters%5B%5D=bisp-collection%3A%22db%22&type=AllFields&filter%5B%5D=%7Eformat%3A%22For-schungsprojekt%22.

[43] GKV-Bündnis für Gesundheit. Bündnisaktivitäten im Überblick [cited 22.05.2025]. Available from: https://www.gkv-buendnis.de/buendnis-aktivitaeten/buendnisaktivitaeten_im_ueberblick/buendnisaktivitae-ten_im_ueberblick_1.html.

[44] Glasgow RE Harden SM Gaglio B Rabin B Smith ML Porter GC. RE-AIM Planning and Evaluation Framework: Adapting to New Science and Practice With a 20-Year Review. Front Public Health. 2019;764. doi:10.3389/fpubh.2019.00064. 10.3389/fpubh.2019.00064 PMC6450067 30984733

[45] Messing S Rütten A. Qualitätskriterien für die Konzipierung, Implementierung und Evaluation von Interventionen zur Bewegungsförderung: Ergebnisse eines State-of-the-Art Reviews. Gesundheitswesen. 2017;79(S 01):S60-S65. ger. doi:10.1055/s-0042-123378. 28399588 10.1055/s-0042-123378

[46] Bauman A Ainsworth BE Bull F Craig CL Hagströmer M Sallis JF. Progress and pitfalls in the use of the International Physical Activity Questionnaire (IPAQ) for adult physical activity surveillance. J Phys Act Health. 2009;6 Suppl 1S5-8. doi:10.1123/jpah.6.s1.s5. 10.1123/jpah.6.s1.s5 19998844

[47] Tcymbal A Messing S Mait R Perez RG Akter T Rakovac I. Validity, reliability, and readability of single-item and short physical activity questionnaires for use in surveillance: A systematic review. PLoS One. 2024;19(3):e0300003. doi:10.1371/journal.pone.0300003 38470871 10.1371/journal.pone.0300003 PMC10931432

[48] World Health Organization (WHO). WHO guidelines on physical activity and sedentary behaviour. Geneva, Switzerland: WHO; 2020. p 93. 33369898

[49] Pedišić Ž Bauman A. Accelerometer-based measures in physical activity surveillance: current practices and issues. Br J Sports Med. 2015;49(4):219–23. doi:10.1136/bjsports-2013-093407. 25370153 10.1136/bjsports-2013-093407

[50] Althoff T Sosič R Hicks JL King AC Delp SL Leskovec J. Large-scale physical activity data reveal worldwide activity inequality. Nature. 2017;547(7663):336–9. doi:10.1038/nature23018. 28693034 10.1038/nature23018 PMC5774986

[51] Dobbins M Husson H DeCorby K LaRocca RL. School-based physical activity programs for promoting physical activity and fitness in children and adolescents aged 6 to 18. Cochrane Database Syst Rev. 2013;2013(2):CD007651.doi:10.1002/14651858.CD007651.pub2. 23450577 10.1002/14651858.CD007651.pub2 PMC7197501

[52] Telama R Yang X Leskinen E Kankaanpää A Hirvensalo M Tammelin T. Tracking of physical activity from early childhood through youth into adulthood. Med Sci Sports Exerc. 2014;46(5):955–62. doi:10.1249/MSS.0000000000000181. 24121247 10.1249/MSS.0000000000000181

[53] Pongiglione B Kern ML Carpentieri JD Schwartz HA Gupta N Goodman A. Do children‘s expectations about future physical activity predict their physical activity in adulthood? Int J Epidemiol. 2020; 49(5):1749–58. doi:10.1093/ije/dyaa131. 33011758 10.1093/ije/dyaa131 PMC7746399

[54] Muller SM. The implications of a fundamental contradiction in advocating randomized trials for policy. World Development. 2020;12710 4831.doi:10.1016/j.worlddev.2019.104831.

[55] Parra JD Edwards DB. Challenging the gold standard consensus: Randomised controlled trials (RCTs) and their pitfalls in evidence-based education. Crit Stud Educ. 2024;65(5):513–30. doi:10.1080/17508487.2024.2314118

[56] Verein Programm Klasse2000 e.V. Klasse2000. 2025 [cited 22.05.2025]. Available from: https://www.klasse2000.de/.

[57] Fit4Future. fit4future – Wir machen Kinder und Jugendliche fit für die Zukunft! 2025 [cited 22.05.2025]. Available from: https://fit-4-future.de.

[58] Helsper N Dippon L Birkholz L Rütten A Kohler S Weber P. What makes community-based, multilevel physical activity promotion last? A systematic review with narrative synthesis on factors for sustainable implementation. Perspect Public Health. 2023;175791392 31186693. doi:10.1177/17579139231186693. 10.1177/17579139231186693 PMC12457715 37539694

[59] Reis RS Salvo D Ogilvie D Lambert EV Goenka S Brownson RC. Scaling up physical activity interventions worldwide: stepping up to larger and smarter approaches to get people moving. Lancet. 2016;388(10051):1337–48. doi:10.1016/ S0140-6736(16)30728-0. 27475273 10.1016/S0140-6736(16)30728-0 PMC5193005

[60] Krupp S Kasper J Hermes A Balck F Ralf C Schmidt T. Das „Lübecker Modell Bewegungswelten“ – Ergebnisse der Effektevaluation. Bundesgesundheitsbl. 2019;62(3):274–81. ger. doi:10.1007/s00103-019-02881-6. 10.1007/s00103-019-02881-6 30729993

[61] Bundesinstitut für Öffentliche Gesundheit. LMB-Angebote [cited 22.05.2025]. Available from: https://www.gesund-aktiv-aelter-werden.de/bewegung/luebecker-modell-bewegungswelten/lmb-angebote/.

[62] Ammann C Atzinger S Froböse I. Das AlltagsTrainingsProgramm (ATP). Public Health Forum. 2017;25(2):165–8. doi:10.1515/pubhef-2016-2157.

[63] Bundesinstitut für Öffentliche Gesundheit. AlltagsTrainingsProgramm (ATP) [cited 26.09.2025]. Available from: https://www.gesund-aktiv-aelter-werden.de/bewegung/alltagstrainingsprogramm-atp/.

[64] DOSB. Mitgliedsorganisationen. 2025 [updated 22.05.2025]. Available from: https://www.dosb.de/ueber-uns/mitgliedsorganisationen.

[65] Statistisches Bundesamt (Destatis). 1,0 % mehr Schülerinnen und Schüler im Schuljahr 2023/2024. 2025 [cited 22.05.2025]. Available from: https://www.destatis.de/DE/Presse/Pressemitteilungen/2024/03/PD24_101_211.html#:~:text=WIESBADEN%20%E2%80%93%20Im%20Schuljahr%202023%2F2024,an%20Schulen%20des%20Gesundheitswesens%20unterrichtet.

[66] Statistisches Bundesamt (Destatis). Anzahl der jährlichen Arztbesuche pro Kopf in Deutschland in den Jahren 1991 bis 2022. 2024 [cited 22.05.2025]. Available from: https://de.statista.com/statistik/daten/studie/77182/umfrage/deutschland-jaehrliche-arztbesuche-pro-kopf-seit-1991/.

[67] Rütten A Frahsa A Abel T Bergmann M de Leeuw E Hunter D. Co-producing active lifestyles as whole-system-approach: theory, intervention and knowledge-to-action implications. Health Promot Int. 2019;34(1):47–59. doi:10.1093/heapro/dax053. 28973298 10.1093/heapro/dax053

[68] Gelius P Brandl-Bredenbeck HP Hassel H Loss J Sygusch R Tittlbach S. Kooperative Planung von Maßnahmen zur Bewegungsförderung: Neue Wege zur Erweiterung von Handlungsmöglichkeiten – Ergebnisse aus dem Forschungsverbund Capital4Health. Bundesgesundheitsbl. 2021;64(2):187–98. ger. doi:10.1007/s00103-020-03263-z. 10.1007/s00103-020-03263-z PMC7843529 33315164

[69] Popp J Grüne E Carl J Semrau J Pfeifer K. Co-creating physical activity interventions: Findings from a multiple case study using mixed methods. Front Public Health. 2022;10975638. doi:10.3389/fpubh.2022.975638. 10.3389/fpubh.2022.975638 PMC9534180 36211644

[70] Hahn LS Thiel A Dembeck V Haigis D Matting L Pomiersky R. Addressing organizational learning to increase readiness for physical activity promotion in seven German nursing homes. PLoS One. 2025;20(5):e0315241.doi:10.1371/journal.pone.0315241. 40354493 10.1371/journal.pone.0315241

[71] Pratt M Varela AR Bauman A. The Physical Activity Policy to Practice Disconnect. J Phys Act Health. 2023;20(6):461–4. doi:10.1123/jpah.2023-0071. 36928002 10.1123/jpah.2023-0071

[72] Bundesministerium für Gesundheit. Konsenspapier. Runder Tisch Bewegung und Gesundheit: Ergebnisse des sektorenübergreifenden Dialogs zur Stärkung der Bewegungsförderung in Deutschland. 2024 [cited 26.09.2025]. Available from: https://www.bundesgesundheitsministerium.de/fileadmin/Dateien/5_Publikationen/Praevention/Broschueren/Konsenspapier_Runder_Tisch.pdf.

[73] Bundesministerium für Forschung, Technologie und Raumfahrt. IMPAQT – Verbesserung von Politik zur Bewegungsförderung und ihrer Auswirkungen auf die gesundheitliche Chancengleichheit. 2024 [cited 07.11.2025]. Available from: https://www.gesundheitsforschung-bmftr.de/de/impaqt-verbesserung-von-politik-zur-bewegungsforderung-und-ihrer-auswirkungen-auf-die-18478.php.

[74] Rütten A Semrau J Helsper N Dippon L Kohler S Pfeifer K. Researchers as Policy Entrepreneurs for Structural Change: Interactive Research for Promoting Processes Towards Health Equity. In: Potvin L Jourdan D, editors. Global Handbook of Health Promotion Research, Vol. 1. Cham: Springer International Publishing; 2022. p. 675–92.

[75] Franks RP Bory CT. Who Supports the Successful Implementation and Sustainability of Evidence-Based Practices? Defining and Understanding the Roles of Intermediary and Purveyor Organizations. New Dir Child Adolesc Dev. 2015;2015(149):41–56. doi:10.1002/cad.20112. 10.1002/cad.20112 26375190

